# Supplementary material for: Leveraging the Mendelian disorders of the epigenetic machinery to systematically map functional epigenetic variation
Source: eLife. 2021 Aug 31;10:e65884. doi: 10.7554/eLife.65884 (PMC8443249; doi:10.7554/eLife.65884)
Supplement: Transparent reporting form [file elife-65884-transrepform1.docx]

***eLife’s* transparent reporting form**

We encourage authors to provide detailed information *within their submission* to facilitate the interpretation and replication of experiments. Authors can upload supporting documentation to indicate the use of appropriate reporting guidelines for health-related research (see [EQUATOR Network](http://www.equator-network.org/%20)), life science research (see the [BioSharing Information Resource](https://biosharing.org/)), or the [ARRIVE guidelines](http://www.plosbiology.org/article/info:doi/10.1371/journal.pbio.1000412) for reporting work involving animal research. Where applicable, authors should refer to any relevant reporting standards documents in this form.

If you have any questions, please consult our Journal Policies and/or contact us: [editorial@elifesciences.org](mailto:editorial@elifesciences.org).

**Sample-size estimation**

- You should state whether an appropriate sample size was computed when the study was being designed
- You should state the statistical method of sample size computation and any required assumptions
- If no explicit power analysis was used, you should describe how you decided what sample (replicate) size (number) to use

Please outline where this information can be found within the submission (e.g., sections or figure legends), or explain why this information doesn’t apply to your submission:

For RNA-Seq experiments we performed the following power calculation using the RNASeqPower package in R. Assuming an alpha of 0.05 and power (1-beta) of 0.8, as well as a low coefficient of variation of counts (0.1) given usage of fully backcrossed inbred mice and an even number of cases of and controls our simulation shows the number of mice needed to detect expression differences of 1.25x, 1.5x, 1.75x, and 2x between two genotypes. For highly expressed genes (>25 reads covering a gene), six animals per genotype should be adequate to detect expression differences of 1.5-fold between the genotypes. Similarly, for moderately expressed genes (5-25 reads covering a gene), six animals per group should be able to detect differences of around 2-fold between the genotypes and thus we aimed to have at least six animals per group. For ATAC-Seq it is harder to perform power calculations and thus here we aimed for an equal number of animals as in RNA-Seq. For each mutant vs wild-type comparison, we sought to match mice with respect to age and sex. The final sample size was also affected by the availability of mice in each cohort (this is always the case for mouse studies) and number able to process simultaneously and ended up with a range 5-12 mice/study. We feel confident that the cohorts in our study equal or exceed what has been seen in similar studies in the literature.

**Replicates**

- You should report how often each experiment was performed
- You should include a definition of biological versus technical replication
- The data obtained should be provided and sufficient information should be provided to indicate the number of independent biological and/or technical replicates
- If you encountered any outliers, you should describe how these were handled
- Criteria for exclusion/inclusion of data should be clearly stated
- High-throughput sequence data should be uploaded before submission, with a private link for reviewers provided (these are available from both GEO and ArrayExpress)

Please outline where this information can be found within the submission (e.g., sections or figure legends), or explain why this information doesn’t apply to your submission:

KS1 and KS2 samples include multiple cohorts, RTS1 is a single cohort due to the availability of the mice (very difficult to breed this model). For all assays, each biological replicate corresponds to a different mouse. There are no technical replicates for these samples. ATAC-Seq and RNA-Seq samples were filtered for exclusion based on sequencing quality, all passing data points were handled in the same way, and no outliers were excluded. The number of independent biological replicates (in our case, individual mice) for ATAC-Seq is stated in the results section “Genome-wide chromatin accessibility profiling reveals extensive overlap between the epigenetic aberrations of the three MDEMs” and is shown in Figures 2e. For RNA-Seq the number of biological replicates is shown in Figures 4e. All high-throughput data have been deposited to GEO (accession GSE162181).

For IgA measurements, technical duplicates were repeat measures of the same animal on the measurement plate (averages were reported), and the IgA serum measurement data are available as Figure 5-Supplemental Table 3. The number of biological replicates is shown in Figures 5e.

**Statistical reporting**

- Statistical analysis methods should be described and justified
- Raw data should be presented in figures whenever informative to do so (typically when N per group is less than 10)
- For each experiment, you should identify the statistical tests used, exact values of N, definitions of center, methods of multiple test correction, and dispersion and precision measures (e.g., mean, median, SD, SEM, confidence intervals; and, for the major substantive results, a measure of effect size (e.g., Pearson's r, Cohen's d)
- Report exact p-values wherever possible alongside the summary statistics and 95% confidence intervals. These should be reported for all key questions and not only when the p-value is less than 0.05.

Please outline where this information can be found within the submission (e.g., sections or figure legends), or explain why this information doesn’t apply to your submission:

All statistical methods used are described in detail in the Methods sections “ATAC-Seq differential analysis”, “RNA-Seq mapping and differential analysis”, and “Testing for statistically significant overlap between two lists of differential features and identifying the common hits”. They are also stated in the relevant places in the Results sections. Exact p-values have been reported, except when the p-value was less than 2.2e-16. In such cases, we followed the standard convention in R and reported “p<2.2e-16”.

(For large datasets, or papers with a very large number of statistical tests, you may upload a single table file with tests, Ns, etc., with reference to sections in the manuscript.)

**Group allocation**

- Indicate how samples were allocated into experimental groups (in the case of clinical studies, please specify allocation to treatment method); if randomization was used, please also state if restricted randomization was applied
- Indicate if masking was used during group allocation, data collection and/or data analysis

Please outline where this information can be found within the submission (e.g., sections or figure legends), or explain why this information doesn’t apply to your submission:

We have no treatment in this study except mice were aged to 2.5-3.5 months under standard conditions according to our University protocol. In these experiments our variable of interest is the genotype of the mice. Samples were taken in groups where wildtype and mutant mice from the same cohort were processed together.

**Additional data files (“source data”)**

- We encourage you to upload relevant additional data files, such as numerical data that are represented as a graph in a figure, or as a summary table
- Where provided, these should be in the most useful format, and they can be uploaded as “Source data” files linked to a main figure or table
- Include model definition files including the full list of parameters used
- Include code used for data analysis (e.g., R, MatLab)
- Avoid stating that data files are “available upon request”

Please indicate the figures or tables for which source data files have been provided:

We have provided 17 supplemental data files, with explicit descriptions for each. All code used for the analyses is available on github (<https://github.com/hansenlab/mdem_overlap>).
